# Learning Environments and Brain Health: A Neuroeducational Perspective

**DOI:** 10.1523/ENEURO.0036-26.2026

**Published:** 2026-03-20

**Authors:** Sadaf Ahmed

**Affiliations:** Psychophysiology Research Unit, Department of Physiology, University of Karachi, Karachi 75270, Pakistan

**Keywords:** brain health, cognitive development, learning environments, neuroplasticity, science education, stress regulation

## Abstract

Science education is traditionally framed as a driver of scientific literacy and economic growth. However, emerging evidence suggests that it may also function as a contributor to public health by shaping brain health across the lifespan. In this invited commentary, I synthesize findings from human and animal studies to examine how enriched, inquiry-based educational experiences intersect with neural processes underlying cognitive development, stress regulation, executive function, and social-emotional well-being. This synthesis is guided by the principle of cognitive compassion, which emphasizes the design of learning environments that support both cognitive and emotional needs. Research on neuroplasticity, stress biology, and motivation indicates that learning contexts characterized by curiosity, emotional safety, and active engagement are associated with adaptive neural function and long-term cognitive resilience. Drawing on empirical literature and illustrative translational observations from educational and community science contexts, I propose that science education can be conceptualized as a population-level contributor to brain health. Framing education through a brain health lens has implications for educational policy, teacher professional development, and public investment in learning environments, particularly in underserved settings. This perspective positions education not only as a mechanism for knowledge transmission but also as a modifiable environmental factor that supports neural and societal resilience.

## Introduction

Traditionally, science education has often been framed in the context of economic growth and technological innovation. However, a complementary perspective positions science instruction as an important contributor to lifelong brain health. Research on classroom practice and developmental learning environments supports the use of enriched, inquiry-based approaches that stimulate neural systems involved in attention, motivation, learning processes, and emotional engagement that facilitate deeper cognitive involvement (Kallery et al., 2022; Dubinsky and Hamid, 2024). Initial exposure to scientific inquiry, e.g., practical exploration or problem-solving exercises, can cause the feeling of curiosity and psychological safety, which are proven to promote learning. In this respect, educational quality not only implies relevant effects on academic performance but also on long-term cognitive and emotional performance at the level of population.

**Figure 1.**
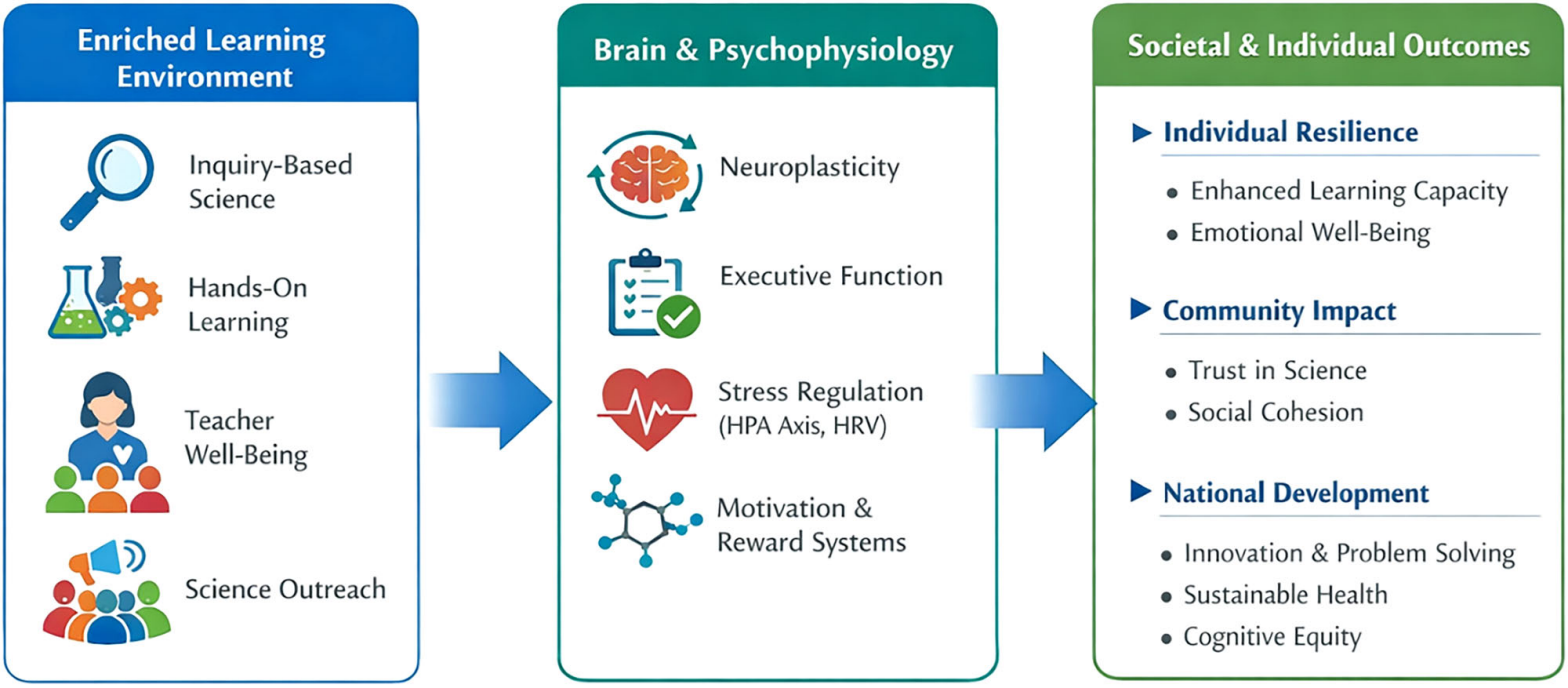
Conceptual framework illustrating how enriched, inquiry-based science education may influence brain health across individual, educational, and societal levels. This conceptual model depicts hypothesized relationships between learning environments and executive, affective, and stress-regulatory neural systems. This figure represents a conceptual integration of existing literature and does not depict experimentally validated pathways or intervention effects.

This view is reinforced by emerging convergent findings provided by neuroscience, psychophysiology, and developmental science that learning environments can cause changes in neural systems associated with attention, memory, motivation, and stress management (Imad, 2022; Zadina, 2023). Characteristics typically linked to enriched science education, such as inquiry-based activity, exploration, and supportive classroom climates, are associated with the engagement of executive control networks, memory-related systems, and stress-regulatory processes (Gruber et al., 2014). These associations as summarized in Table 1 indicate that learning environments are not passive settings of learning process but an active developmental input that has cognitive and social-emotional functional implications. Figure 1 shows a conceptual model of how enriched science education can have relationships with executive, reward-related, and stress-regulatory systems with time.

**Table 1.** Science education as brain health policy: conceptual links

Educational practice	Primary neural systems engaged	Individual-level outcomes	Societal-level implications
Inquiry-based science learning	Prefrontal executive networks; hippocampal memory systems	Improved attention regulation, cognitive flexibility, and problem-solving	Enhanced analytical reasoning, innovation, and informed decision-making
Hands-on experimentation and play	Multisensory integration; reward-related circuits	Increased motivation, engagement, and memory consolidation	Sustained participation in STEM learning
Teacher well-being and self-regulation	Autonomic regulation; reduced stress reactivity	Calmer classroom climate and improved emotional regulation	Reduced teacher burnout; more sustainable educational systems
Science outreach and public engagement	Social-affective and reward networks	Greater sense of agency and reduced stress reactivity	Increased public trust in science and community resilience
Early exposure to science learning	Experience-dependent neuroplasticity	Foundational executive function and long-term cognitive resilience	Intergenerational gains in educational attainment and human capital

In the following sections, I discuss four areas of brain wellness that are especially pertinent to education: neural development, stress regulation, executive function, and social-emotional well-being. This commentary relies on the existing empirical research and examples in classrooms and communities to suggest that the investment in high-quality science education is consistent with the overall objectives of the population health. All the way, real-world examples have been brought out as observational and contextual but not as experimentally proved interventions and this is meant to base theoretical formulations in educational practice but not to confuse education and clinical science.

## Neural Development and Enriched Learning

Neuroscience has been able to clearly show that the development of the brain is heavily influenced by experience especially in childhood. According to Kolb and Gibb (2011), brain maturation is the outcome of dynamic interplay between genetic factors and environmental input with enriched ones (adopted synaptic development and network integration) caring of sensory, social, and cognitive stimulation (Mualem et al., 2024). Conversely, these processes can be impaired by chronically stressful or deprived conditions. Animal models demonstrate that experience-dependent plasticity in the brain can be characterized by a greater density of synapses and complexity of their structure under a condition of enrichment (Hille et al., 2024).

Educational enrichment in humans has been linked with signs of healthy cognitive development and cognitive resilience in the long term. Problem-solving and scientific inquiry help in enhancing the strength of the already existing neural connections and foster the development of new neural connections, which can be referred to as cognitive reserve. According to Stern (2012), the ability of the brain to endure age-related or disease-related alteration is boosted by a long-term intellectual activity throughout the lifespan. Other epidemiological research also shows that there is a lower risk of dementia with increased education, which has often been credited with reserve-related mechanisms (Alvares Pereira et al., 2022).

Community-based science outreach observations provide illustrative examples of how enriched learning situations may facilitate attention and problem-solving in children; however, these observations are descriptive and should not be interpreted as controlled evaluations. In practical tasks involving simple construction and observation, teachers informally reported greater persistence and engagement; these accounts are observational and serve to contextualize the broader literature on experience-dependent plasticity.

The problem of equity is also prefigured by the perception of education through the prism of brain health. Children raised in low-resource settings will be more prone to living in chronic stress and lack of access to cognitively enriching experience, which have been linked to an altered neurodevelopmental pathway (Nelson et al., 2025). The scientific study concerning toxic stress shows that the extended activation of stress response systems in a sensitive part of the development stage may disrupt neural growth and functioning of the executive. Public health-wise, the potential solution to such disparities is the ability to increase the access to enhanced, inquiry-based learning experiences, which would facilitate adaptive brain development among populations. Here, educational investment serves as a long-term sustenance of neural and cognitive strength instead of being a tool to improve educational progress.

## The Stress Regulation and Learning Environments

Learning takes place in the environment of physiological and emotional conditions of the students with some fluctuations in stress and arousal. Stress biology studies reveal that chronic stress-related arousal may result in poor performance of cognitive processes vital to learning, including working memory, attentional control, and self-regulation. According to McEwen and Gianaros (2011), stress exposure is regulated and changed over time on the brain, whereas acute stresses can be adaptive, long-lasting stress that can be linked to impairments in the functions of the brain systems of executive control and threat processing. Such processes are observed in the high-stress educational settings where students who suffer continuous adversity may fail to maintain attention and engagement.

The stress regulation processes in relation to attention and memory may be assisted by the learning environments that involve emotional safety, predictability, and supportive relationships. Activities based on inquiry that are applied in such settings can facilitate concentrated engagement states that are in accordance with low cognitive interference due to stress. The brief reflection or self-regulation practices implemented in classroom contexts have been associated in some studies with improvements attentional climate, and teacher professional development settings have been observed to have better classroom environments when teachers self-regulate. These classroom-level observations have not been evaluated through controlled intervention trials and are presented here as contextual illustrations consistent with existing stress and regulation research (Maricut´oiu et al., 2023).

The policy is best interpreted as a part of the learning preparedness instead of a direct education result. Schools should not be clinical, but nonclinical indicators, including student engagement, perseverance on demanding tasks, classroom environment, and attendance, can be practicable indications of encouraging learning conditions. Creating education systems that limit avoidable stressors and promote adaptive control, thus, can improve academic learning and long-term well-being, without repositioning schools as mental health interventions.

## Inquiry, Curiosity, and Executive Function

Social and emotional well-being are also supported by science education as science education involves a social nature of learning. The trust, empathy, and a sense of belonging can be cultivated by moments of common inquiry, like joint experiments, or solving a problem together. Social and affective neuroscience literature suggests that cooperative learning activates brain circuits of social bonding, reward, and stress regulation, which facilitate emotional safety and long-term engagement. Science activities provided on the community level also provide settings to joint discovery that can enhance social relationships and belief in the process of science. Science outreach can thus be a platform to re-establish cooperative interaction and mutual curiosity in an environment that is characterized by social fragmentation or distrust of expertise (Zurn and Bassett, 2022).

This has been seen to have not only pertinent impacts in community outreach but also in classroom culture. Instructional and teaching conditions that encourage questioning, ambiguity, and error-making assist students in controlling their emotions when carrying out cognitively challenging activities. Learners who practice managing excitement, frustration, and uncertainty during inquiry-based learning may develop transferable regulatory skills, though this extrapolation remains theoretically grounded rather than empirically tested within the present framework. The role of teacher well-being is crucially important in the process since the ability of educators to self-regulate and show empathy creates positive classroom climates (Jennings et al., 2019). Collectively, these practices lead to what can be termed as a culture of cognitive compassion where intellectual challenge and emotional support are mutually strengthening as opposed to being mutually exclusive priorities.

## Social-Emotional Well-Being and Science in Community Bond

Social and emotional well-being are also supported through science education because learning science frequently occurs within collaborative and socially interactive environments that foster emotional meaning-making and engagement (Immordino-Yang, 2016). There can be moments of mutual inquiry, e.g., joint experiments or joint problem-solving that can lead to a sense of trust, empathy, and belief. Social and affective neuroscience studies show that collaborative learning activates social bonding, reward, and stress management systems that aid in emotional safety and continued interest (Huang and Lajoie, 2023). Science activities conducted within a community also establish settings in which mutual learning can occur and possibly enhance social bonds and trust between science and the community. Science outreach can thus serve as a context of restoring cooperative practice and mutual interest in the environments characterized by social fragmentation or distrust toward expertise.

These impacts not only manifest in community outreach but they are also reflected in classroom culture. Emotional regulation of students during cognitively challenging tasks is facilitated by learning environments that encourage questioning, uncertainty, and mistakes making. Learners that learn how to handle excitement, frustration, and uncertainty during inquiry-based learning might be more likely to control emotions outside the classroom. The well-being of the teacher is central to this process because the ability to use empathy and self-regulation to create a supportive classroom environment is determined by an educator (Jennings et al., 2019). These practices combined can be termed as a culture of cognitive compassion where intellectual stimulation and emotional nurturing do not act as competing interests but as complimentary concerns.

## Science Education as Brain Health Policy

There are significant policy and practice implications of the notion of science education as a means of brain health. The rationale of considering education as a population-relevant context of brain health throughout life is supported by evidence of links between enriched learning conditions and neural mechanisms involved in attention, stress regulation, and executive functional aspects. In this sense, the cognitive as well as emotional resources of a society are influenced significantly in school, and this is what influences the closer correlation of education and the priorities of health in the public. The educational policy could thus integrate the well-being indicators with the achievement in academics, such as student engagement, classroom climate, and persistence in doing cognitively challenging tasks without redefining the schools as clinics.

Instead of using distinct interventions, scalability of science education as brain health policy means organized interaction between educational and health systems. Education and health ministries can facilitate joint professional development that helps teachers to have a basic knowledge of stress management, attention, and inquiry-based learning and uphold the lines between teaching and clinical interventions (Whiting et al., 2021). Responsibility in these partnerships can be based on aggregate, nonclinical measures of learning readiness like attendance records, participation, and classroom atmosphere and position cross-sector coordination as a governance model of matching educational practice with population-level brain health objectives as opposed to an intervention trial.

Teacher preparation investment is one of the vital aspects of sustainability. Neuroscience-informed practice does not expect teachers to become neuroscientists, but they should be knowledgeable about the interaction between attention, stress, curiosity, and emotion and learning (Dubinsky et al., 2022). Professional development, based on practice oriented inquiry design and classroom routine, is more likely to result in lasting change as compared with information laden training. International cooperation also promotes scalability because it facilitates an exchange of context-appropriate strategies between educational systems. Collaborations between researchers, educators, and community groups, e.g., the ones created by international neuroscience and education networks exemplify how knowledge of research can be applied in locally meaningful ways. Government-funded science outreach, informal learning environments, therefore, is a viable equity-based strategy to promote population-level cognitive resilience and life-long learning.

## Conclusion: From Principle to Practice

**Figure 2.**
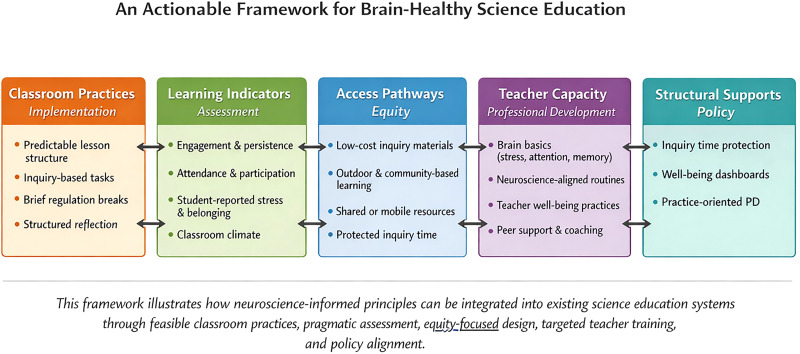
Conceptual framework for integrating neuroscience-informed principles into existing science education systems. The framework outlines feasible classroom practices, pragmatic assessment approaches, equity-oriented design, targeted teacher professional development, and policy alignment. This framework is presented as a translational heuristic and should not be interpreted as an evidence-based intervention model or implementation trial.

When science education is perceived as a determinant of the health of the brain, it is not necessary to restructure the systems in order to translate it to practice but to refine the available systems. Routine classroom procedures, inquiry-based problem-solving, and short periods of self-regulation can be integrated into regular classroom science instruction to facilitate attentional regulation, intellectual flexibility, as well as engagement. These outcomes do not require clinical assessment; feasible outcomes including perseverance in activities involving cognitively challenging challenges, classroom atmosphere, attendance, and student self-reported interest can be plausible indicators of learning readiness at any given time. Equity also demands that enriched learning environments should not rely on expensive infrastructure and inquiry-based science should rely on the locally available materials and a community collaboration providing scalable opportunities in the under-resourced environment. Sustainability rests on teacher preparation based on some bottom-line knowledge on stress regulation, attention, and inquiry design, with the support of policy alignment that secures inquiry time, and incorporation of indicators of well-being into school improvement cycles as shown in Figure 2.

Lastly, the framework that is developed in this case is based on a concept that can be defined as cognitive compassion: the deliberate structure of learning conditions that would consider the cognitive and emotional needs of learners. These settings do not water down scientific rigor but are instead consistent with the inbuilt sense of being driven by the brain toward meaning, mastery and exploration, which favors resilience and adaptability in uncertain circumstances. The results, including curiosity, a sense of belonging, and being comfortable with failure, are not readily measurable using standardized measures; however, they are important indicators of healthy cognitive and emotional growth that are important in education and the general health of the population.

## Synthesis

Reviewing Editor: Christophe Bernard, INSERM &Institut de Neurosciences des Systèmes Decisions are customarily a result of the Reviewing Editor and the peer reviewers coming together and discussing their recommendations until a consensus is reached. When revisions are invited, a fact-based synthesis statement explaining their decision and outlining what is needed to prepare a revision will be listed below. The following reviewer(s) agreed to reveal their identity: NONE.

To further strengthen the manuscript, I suggest minor revisions focused on clarity rather than content. Specifically, it would be helpful to (1) slightly sharpen language in places where empirical evidence transitions into illustrative or observational examples, ensuring readers consistently understand the conceptual scope of the claims; and (2) briefly reiterate in the figure captions and/or discussion that the presented frameworks are theoretical syntheses rather than evaluated intervention models, to avoid over-interpretation by non-specialist readers.

## Author Response

Response to Reviewers Manuscript Title: Learning Environments and Brain Health: A Neuroeducational Perspective Author: Sadaf Ahmed Manuscript ID: eN-COM-0036-26R1 General Response We thank the Editor and Reviewer for the constructive feedback. In response, we have implemented minor revisions to sharpen conceptual clarity, particularly regarding transitions between empirical findings and illustrative examples. We have also strengthened language in the figures and discussion to clarify that the presented frameworks represent theoretical syntheses rather than evaluated intervention models. These revisions enhance interpretive precision without altering the manuscript's central thesis.

Reviewer Comment The manuscript would benefit from clearer distinctions between empirical evidence and illustrative observations, and from reiterating that the figures represent theoretical frameworks rather than intervention models.

Response We appreciate this suggestion and have revised the manuscript accordingly.

1. Empirical vs. Illustrative Distinction o Softened causal phrasing (e.g., replacing stronger language with "associated" or "illustrative"). o Clarified that community outreach and classroom examples are descriptive and not experimentally evaluated. o Added boundary language where extrapolations extend beyond directly tested findings.

2. Figure Clarifications o Revised Figure 1 caption to specify that it reflects conceptual integration rather than validated pathways. o Revised Figure 2 caption to clarify that the framework is a translational heuristic, not an evidence-based intervention model.

3. Discussion and Conclusion o Added explicit statements noting that the manuscript synthesizes convergent empirical literature and does not present science education as a clinical or public health intervention.

We believe these revisions address the Reviewer's concerns by strengthening conceptual boundaries and preventing over-interpretation, particularly by non-specialist readers.

We are grateful for the opportunity to improve the manuscript.
